# Sun Safety Intervention for Elementary School Students

**DOI:** 10.1111/pde.70234

**Published:** 2026-05-10

**Authors:** Camille Moeckel, Rachel Anderson, Laurie Krnajic, Amy Longenecker, Elizabeth M. Billingsley, Andrea L. Zaenglein, Charlene Lam

**Affiliations:** ^1^ Penn State College of Medicine Hershey Pennsylvania USA; ^2^ Clinical Trials Department Penn State Health Hershey Pennsylvania USA; ^3^ Department of Dermatology Penn State Health Milton S. Hershey Medical Center Hershey Pennsylvania USA; ^4^ Penn State Cancer Institute, Penn State Milton S. Hershey Medical Center Hershey Pennsylvania USA

**Keywords:** child behavior, health education, health promotion, school‐based intervention, skin neoplasms, sun protection

## Abstract

This study evaluated the impact of an elementary school‐based intervention that aimed to improve sun safety knowledge and behaviors among third‐ and fourth‐grade students in Pennsylvania through an educational video, t‐shirt design contest, and interactive assembly. From pre‐survey (*n* = 88) to post‐survey (*n* = 86), students showed significant improvements in awareness of the link between sun exposure and skin cancer (*p* < 0.001). Between pre‐survey and 6‐month follow‐up (*n* = 67), no significant changes were found in reported sunscreen use and reapplication, hat‐wearing, or sunburn incidence, but knowledge of skin cancer risk from the sun remained high (*p* < 0.001). In summary, the intervention significantly increased awareness of the link between sun exposure and skin cancer, with knowledge remaining high at 6‐month follow‐up, but sustained behavioral change was not observed, suggesting a need for continued reinforcement or different intervention.

## 1 Introduction

Ultraviolet radiation (UVR) exposure during childhood significantly contributes to lifetime skin cancer risk, making early sun protection critical. Experiencing five or more severe sunburns between ages 15 and 20 years is strongly associated with both non‐melanoma and melanoma skin cancers, with melanoma risk specifically increased by 80% [1]. Therefore, elementary‐aged children are an important target for sun safety interventions. School‐based programs have reported increases in knowledge about sun safety and skin cancer but found sustained behavioral change challenging [2, 3, 4, 5]. This study evaluated the impact of Penn State Health Dermatology's “Sun Protection Hero” intervention on third‐ and fourth‐grade students' sun safety knowledge and behaviors, assessed through pre‐, immediate post‐, and 6‐month follow‐up surveys.

## 2 Methods

The intervention was conducted at a Title I urban public elementary school in Lebanon, Pennsylvania, that serves roughly 370 kindergarten through fifth‐grade students (Hispanic 71.6%, White 20.5%, Black 4.1%). It began with a kickoff event led by the dermatology research team, during which third‐ and fourth‐grade students with parental consent completed a pre‐survey (*n* = 88), watched an educational video on sun protection created by a nurse practitioner and two pediatric dermatologists, asked questions, and completed an immediate post‐survey (*n* = 86). Written materials, including parental consent forms, were provided in both English and Spanish. Students then had 2 weeks to submit drawings for a “Sun Protection Hero” t‐shirt design contest, with school voting selecting six finalists and Penn State's Dermatology Department choosing the winner. Roughly 14 weeks after the initial kickoff event, a 30‐min assembly led by medical professionals was held to announce the winner (Figure 1) and included a brief educational segment, interactive quiz, and skit. Six‐month follow‐up surveys were completed by 67 students. Of the pre‐survey sample, two students (2.3%) did not complete the immediate post‐survey, and 21 (23.9%) were lost at the 6‐month follow‐up due to relocation or scheduling conflicts.

**FIGURE 1:**
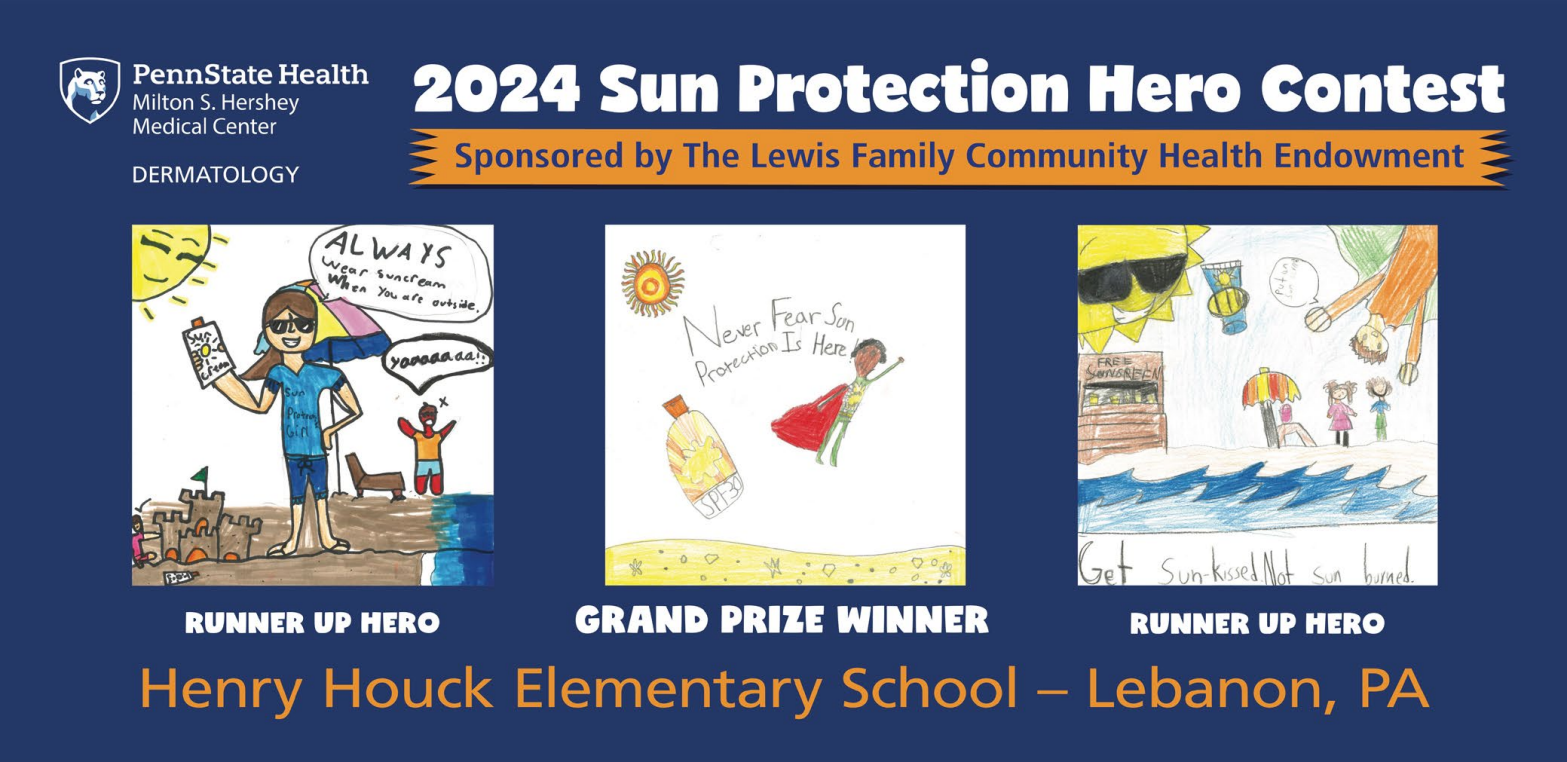
Results of t‐shirt design contest in which students were invited to create a superhero‐themed design promoting sun safety.

Each survey included 10 identical questions: 3 assessing demographics (age, grade, skin tone) and 7 “yes” or “no” questions about outdoor activity on sunny days, sunscreen and hat use, sunburn history, and knowledge of the sun exposure‐skin cancer link and sunscreen's purpose (Data S1). Skin tone was assessed using the Self‐Assessment Skin Tone Palette (SASTP), an objective and validated 9‐point scale where students compared their inner forearm to a color palette ranging from the lightest shade (A1) to the darkest shade (C3).

Data were aggregated via REDCap (Research Electronic Data Capture) and analyzed using SPSS (Version 30.0.0.0). Descriptive statistics and z‐tests comparing proportions of “yes” responses between surveys (α = 0.05) were utilized.

## 3 Results

Pre‐ and post‐survey comparisons showed no significant change in reports of playing outside on sunny days (94.3% vs. 93.0%, *p* = 0.726) (Figure 2). Reports of hat use increased insignificantly (38.6% vs. 50.0%, *p* = 0.131), while reports of sunscreen use (59.1% vs. 73.3%, *p* = 0.049) and reapplication (23.9% vs. 45.3%, *p* = 0.003) increased significantly. Reported sunburns in the past year increased insignificantly (42.0% vs. 48.8%, *p* = 0.368). Awareness of the sun exposure‐skin cancer link rose significantly (55.7% vs. 82.6%, *p* < 0.001), while understanding sunscreen's purpose increased slightly but insignificantly (86.4% vs. 94.2%, *p* = 0.082).

**FIGURE 2:**
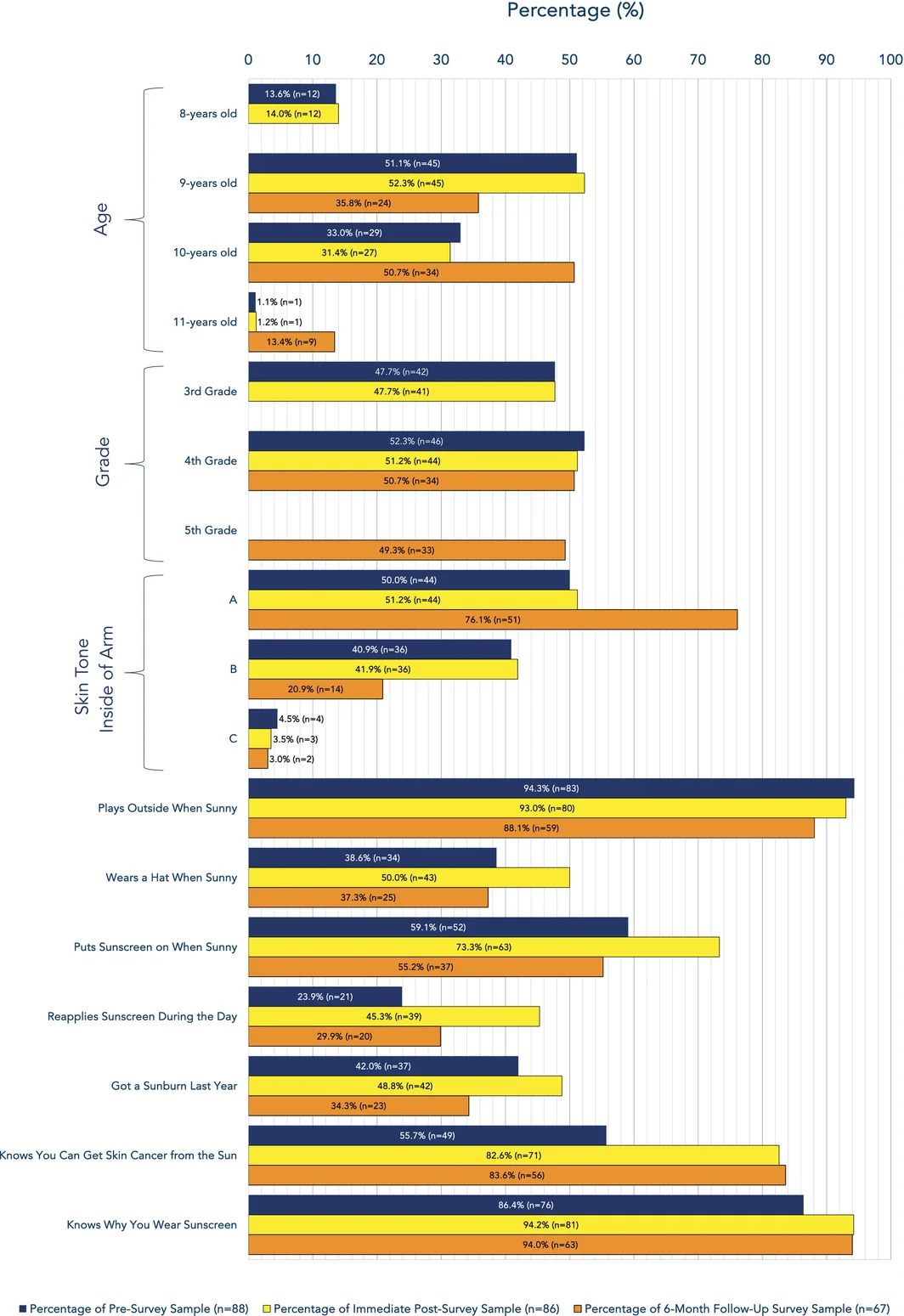
Survey responses from pre‐survey (*n* = 88), immediate post‐survey (*n* = 86), and 6‐month follow‐up samples (*n* = 67). At baseline, most students were 9–10 years old and in third or fourth grade; at 6‐month follow‐up, ages were 9–11, with grades roughly split between fourth and fifth. The majority of participants reported having skin tone A, followed by B. Between pre‐ and immediate post‐surveys, there was a statistically significant increase in respondents who reported applying sunscreen (59.1% vs. 73.3%, *p* < 0.05) and reapplying sunscreen (23.9% vs. 45.3%, *p* < 0.01). Knowledge that sun exposure can cause skin cancer rose significantly from pre‐survey both immediately following the intervention at the post‐survey (55.7% vs. 82.6%, *p* < 0.001) and at 6‐month follow‐up (55.7% vs. 83.6%, *p* < 0.001).

At 6‐month follow‐up, no significant behavior changes were observed compared to the pre‐survey. Reports of playing outside (94.3% vs. 88.1%, *p* = 0.165), hat use (38.6% vs. 37.3%, *p* = 0.865), and sunscreen use (59.1% vs. 55.2%, *p* = 0.631) declined slightly, while sunscreen reapplication rose modestly (23.9% vs. 29.9%, *p* = 0.401). Also, reports of sunburns in the past year decreased from 42.0% to 34.3%, but insignificantly (*p* = 0.327). Awareness of the sun exposure‐skin cancer link remained significantly higher than baseline (55.7% vs. 83.6%, *p* < 0.001) and understanding sunscreen's purpose rose slightly but insignificantly (86.4% vs. 94.0%, *p* = 0.121).

## 4 Discussion

The intervention significantly increased awareness of the link between sun exposure and skin cancer, with awareness sustained at 6 months. Directly following the intervention, students reported significantly increased sunscreen use and reapplication; as the pre‐ and post‐surveys were completed on the same day, this does not indicate actual behavioral change but does point towards increased knowledge about protective methods and perhaps an initial motivation to adopt behaviors. However, at 6‐month follow‐up, there were no significant changes in behaviors from baseline, and the 7.7% decrease in reported sunburns was insignificant. This disconnect between increased knowledge and lack of behavioral change aligns with prior studies evaluating short‐duration, school‐based programs and highlights the need for ongoing reinforcement [2, 3, 4, 5] or different intervention strategies.

Research supports longer‐term, multi‐unit interventions [6], though even extended programs tend to yield greater improvements in knowledge than behavior [7]. One promising approach for additional reinforcement is leveraging technology, such as texts, emails, or multimedia messaging to students and their families, to increase sun safety education while overcoming time constraints of school‐based health education [4]. Additionally, student “sun safety champions” could model and normalize protective practices. As parental behavior and attitudes influence children's sun safety habits through both direct guidance and observational modeling, future multi‐unit interventions should actively engage caregivers and incorporate strategies to educate and motivate parents to adopt sun protective practices [8, 9].

This study has several limitations that may have introduced bias, including the lack of a control group, small sample size, and 21‐student attrition between pre‐survey and 6‐month follow‐up. The study population was limited to students at one school with parental consent, affecting generalizability, and gender was not collected as a demographic variable, limiting our ability to assess potential differences in photo‐protection behaviors between male and female students. The reliance on self‐reporting by children also represents a limitation, as social desirability bias may have led to over‐reporting of sun safety behaviors, especially immediately post‐intervention. Additionally, seasonal variation may have influenced behavior; the intervention was in late May, while follow‐up was in late November, when sun protection is less frequent. Since seasonality can impact self‐reports of sun protection [10], future studies should schedule follow‐ups during comparable times of year. Also, future studies should consider conducting a preliminary cross‐sectional study to inform intervention design and including an earlier follow‐up survey (e.g., at 3 months) to assess shorter‐term retention of behavior changes.

## 5 Conclusion

This study evaluated a novel, multi‐component intervention that included an interactive competition and a 6‐month follow‐up survey to assess whether changes in sun safety knowledge and behaviors were sustained. These findings about improved knowledge without lasting behavior change highlight the need for ongoing reinforcement strategies and encourage future research to identify effective strategies for promoting long‐term sun protection in children.

## Funding

The intervention and subsequent research were supported by the Lewis Family Community Health Endowment.

## Ethics Statement

Deemed exempt research by the Penn State College of Medicine Institutional Review Board; study ID: IRB STUDY24443.

## Consent

Written informed consent was obtained from the parents or legal guardians of participants in the study. Assent was also obtained from children, in accordance with institutional and ethical guidelines.

## Conflicts of Interest

The authors declare no conflicts of interest.

## Supporting information


**Data S1:** Supporting Information.

## Data Availability

Data is available upon reasonable request to corresponding author.
