# Supplementary material for: Sun Safety Intervention for Elementary School Students
Source: Pediatr Dermatol. 2026 May 10;43(5):1163–6. doi: 10.1111/pde.70234 (PMC13617170; doi:10.1111/pde.70234)
Supplement: Supplementary file 1 [file PDE-43-1163-s001.pdf]

[redacted]

Pre Survey

Record ID ______________________

1. How old are you? ______________________

2. What grade are you in?
   - ◯ 3rd Grade
   - ◯ 4th Grade

3. Circle the color that matches the inside of your arm:
   - ◯ A1
   - ◯ A2
   - ◯ A3
   - ◯ B1
   - ◯ B2
   - ◯ B3
   - ◯ C1
   - ◯ C2
   - ◯ C3

4. When it is sunny out do you play outside?
   - ◯ Yes
   - ◯ No

5. When it is sunny out do you wear a hat?
   - ◯ Yes
   - ◯ No

6. When it is sunny out do you put sunscreen on?
   - ◯ Yes
   - ◯ No

7. Do you put on sunscreen more than one time during the day?
   - ◯ Yes
   - ◯ No

8. Did you get a sunburn last year?
   - ◯ Yes
   - ◯ No

9. Can you get skin cancer from the sun?
   - ◯ Yes
   - ◯ No

10. Do you know why you wear sunscreen?
    - ◯ Yes
    - ◯ No

[redacted]

Post Survey

1. How old are you? ____________________________

2. What grade are you in?
   - ◯ 3rd Grade
   - ◯ 4th Grade

3. Circle the color that matches the inside of your arm:
   - ◯ A1
   - ◯ A2
   - ◯ A3
   - ◯ B1
   - ◯ B2
   - ◯ B3
   - ◯ C1
   - ◯ C2
   - ◯ C3

4. When it is sunny out do you play outside?
   - ◯ Yes
   - ◯ No

5. When it is sunny out do you wear a hat?
   - ◯ Yes
   - ◯ No

6. When it is sunny out do you put sunscreen on?
   - ◯ Yes
   - ◯ No

7. Do you put on sunscreen more than one time during the day?
   - ◯ Yes
   - ◯ No

8. Did you get a sunburn last year?
   - ◯ Yes
   - ◯ No

9. Can you get skin cancer from the sun?
   - ◯ Yes
   - ◯ No

10. Do you know why you wear sunscreen?
    - ◯ Yes
    - ◯ No

[redacted]

[redacted]

6 Month Survey

1. How old are you?

   ____

2. What grade are you in?
   - ◯ 4th Grade
   - ◯ 5th Grade

3. Circle the color that matches the inside of your arm:
   - ◯ A1
   - ◯ A2
   - ◯ A3
   - ◯ B1
   - ◯ B2
   - ◯ B3
   - ◯ C1
   - ◯ C2
   - ◯ C3

4. When it was sunny out did you play outside?
   - ◯ Yes
   - ◯ No

5. When it was sunny out did you wear a hat?
   - ◯ Yes
   - ◯ No

6. When it was sunny out did you put sunscreen on?
   - ◯ Yes
   - ◯ No

7. Did you put on sunscreen more than one time during the day?
   - ◯ Yes
   - ◯ No

8. Did you get a sunburn last year?
   - ◯ Yes
   - ◯ No

9. Can you get skin cancer from the sun?
   - ◯ Yes
   - ◯ No

10. Do you know why you wear sunscreen?
   - ◯ Yes
   - ◯ No
